# A hormone-dependent feedback-loop controls androgen receptor levels by limiting MID1, a novel translation enhancer and promoter of oncogenic signaling

**DOI:** 10.1186/1476-4598-13-146

**Published:** 2014-06-09

**Authors:** Andrea Köhler, Ümmühan Demir, Eva Kickstein, Sybille Krauss, Johanna Aigner, Beatriz Aranda-Orgillés, Antonios I Karagiannidis, Clemens Achmüller, Huajie Bu, Andrea Wunderlich, Michal-Ruth Schweiger, Georg Schaefer, Susann Schweiger, Helmut Klocker, Rainer Schneider

**Affiliations:** 1 Institute of Biochemistry, Center of Molecular Biosciences Innsbruck (CMBI), University of Innsbruck, 6020 Innsbruck, Austria; 2 Department of Urology, Innsbruck Medical University, 6020 Innsbruck, Austria; 3 Max-Planck Institute for Molecular Genetics, 14195 Berlin, Germany; 4 Universitätsmedizin Mainz, Institute for Human Genetics, 55131 Mainz, Germany; 5 Division of Medical Sciences, Medical School, DD1 9SY Dundee, UK; 6 Institute of Vertebrate Genetics, Max-Planck Institute for Molecular Genetics, 14195 Berlin, Germany; 7 Department of Pathology, Innsbruck Medical University, 6020 Innsbruck, Austria; 8 Present address: German Center for Neurodegenerative Diseases (DZNE), Biomedical Center (BMZ1), Building 344, 53127 Bonn, Germany

**Keywords:** Androgen receptor, MID1, Translation, Prostate cancer, Reciprocal regulation

## Abstract

**Background:**

High androgen receptor (AR) level in primary tumour predicts increased prostate cancer (PCa)-specific mortality. Furthermore, activations of the AR, PI3K, mTOR, NFκB and Hedgehog (Hh) signaling pathways are involved in the fatal development of castration-resistant prostate cancer during androgen ablation therapy. MID1, a negative regulator of the tumor-suppressor PP2A, is known to promote PI3K, mTOR, NFκB and Hh signaling. Here we investigate the interaction of MID1 and AR.

**Methods:**

AR and MID1 mRNA and protein levels were measured by qPCR, Western blot and immunohistochemistry. Co-immunoprecipitation followed by PCR and RNA-pull-down followed by Western blot was used to investigate protein-mRNA interaction, chromatin-immunoprecipitation followed by next-generation sequencing for identification of AR chromatin binding sites. AR transcriptional activity and activity of promoter binding sites for AR were analyzed by reporter gene assays. For knockdown or overexpression of proteins of interest prostate cancer cells were transfected with siRNA or expression plasmids, respectively.

**Results:**

The microtubule-associated MID1 protein complex associates with AR mRNA via purine-rich trinucleotide repeats, expansions of which are known to correlate with ataxia and cancer. The level of MID1 directly correlates with the AR protein level in PCa cells. Overexpression of MID1 results in a several fold increase in AR protein and activity without major changes in mRNA-levels, whereas siRNA-triggered knockdown of MID1 mRNA reduces AR-protein levels significantly. Upregulation of AR protein by MID1 occurs via increased translation as no major changes in AR protein stability could be observed. AR on the other hand, regulates MID1 via several functional AR binding sites in the MID1 gene, and, in the presence of androgens, exerts a negative feedback loop on MID1 transcription. Thus, androgen withdrawal increases MID1 and concomitantly AR-protein levels. In line with this, MID1 is significantly over-expressed in PCa in a stage-dependent manner.

**Conclusion:**

Promotion of AR, in addition to enhancement of the Akt-, NFκB-, and Hh-pathways by sustained MID1-upregulation during androgen deprivation therapy provides a powerful proliferative scenario for PCa progression into castration resistance. Thus MID1 represents a novel, multi-faceted player in PCa and a promising target to treat castration resistant prostate cancer.

## Background

The androgen receptor (AR) is the key transcription factor regulating androgen-dependent gene expression and is critical for the development and maintenance of male sexual organs like the prostate. In the adult prostate, survival and function of the secretory epithelia is dependent on continuous androgen stimulation and this cell type is thought to be transformed in prostate adenocarcinoma [1].

Prostate cancer (PCa) is the most common malignancy diagnosed in male humans and the second leading cause of male cancer deaths in Western countries [2]. The two most frequent aberrantly activated signaling pathways found in prostate cancer are controlled by the AR and PI3K [3]. However, the AR is the key regulator and oncogenic driver of progression and therapy resistance in PCa. It is up-regulated in late disease stages by gene amplification and other, non-genomic mechanisms [4].

MID1 is a microtubule-associated ubiquitin E3 ligase, which is mutated in the X-linked inherited disorder Opitz BBB/G syndrome (OS). This syndrome is characterized by mild intellectual disability and malformations of the ventral midline along with ocular hypertelorism (widely spaced eyes) and hypospadias (a frequent birth defect, in which the opening of the urethra is on the underside of the penis). These urogenital malformations resemble those of patients with partial androgen insensitivity syndrome [5], suggesting a link between MID1 and androgen signaling.

A main function of MID1 and its binding partner alpha4 (α4) is to trigger the degradation of the catalytic subunit of the tumor suppressor phosphatase 2A (PP2A) via the ubiquitin proteasome pathway [6,7]. PP2A is a cellular master regulator and an important player in the mTOR pathway, opposing mTOR kinase activity and thereby down-regulating proliferation and cell survival. Recently it was shown that MID1, by inhibiting PP2A, also positively modulates inflammatory pathways through the activation of NFκB [8], a crucial oncogenic factor [9] pivotal for metastasis of PCa [10]. Moreover, PP2A is the principal phosphatase down-regulating cytochrome P450c17 and 17,20 lyase (CYP17A1) activity, resulting in decreased levels of androgens [11]. CYP17A1 is the target of abiraterone acetate, a recently approved new drug for treatment of castration resistant prostate cancer inhibiting androgen biosynthesis [12]. Furthermore, MID1, via downregulation of microtubule-associated PP2A, has a modulatory effect on another major oncogenic pathway, namely Hedgehog signalling: by promoting the nuclear translocation and activity of the microtubule-associated transcription factor GLI3 that positively regulates the oncogenic cyclin D1 [13].

Loss of function mutations of MID1 results in the accumulation of PP2A and the hypophosphorylation of its targets, which in turn results in altered protein functions [6,14]. As a consequence, the increased levels of PP2A disrupt the mTOR/Raptor complex and down-regulate mTORC1 signaling, resulting in reduced S6K1 phosphorylation, cell size decrease, and reduced cap-dependent translation [15].

In addition to its function in the regulation of PP2A, MID1, together with the PP2A subunit α4, represent the core of a large microtubule-bound multiprotein complex that associates with active polyribosomes and mRNAs. This complex associates to microtubules and targets specific mRNAs to MID1 mediated translation [16,17]. The sequence motifs that direct the MID1 complex towards specific mRNAs are purine-rich stem-loop structures [16] and include also expanded trinucleotide repeats as found in mutant huntingtin [18].

Moreover, PDPK-1, an important player of PI3K/Akt and mTOR/PP2A signaling is also translationally up-regulated by the MID1 complex [16]. PDPK-1 is the first node in the PI3K signaling output and activates Akt by phosphorylation. Its overexpression and increased gene copy numbers are common events found in cancer [17]. In contrast, down-regulation of PDPK-1 levels inhibits migration and experimental metastasis [19]. In conclusion, these findings identify MID1 as an interesting novel upstream modulator of proliferating pathways and as an important regulatory hub in tumor cells, making it a promising target for anti-cancer drug development.

The androgen receptor mRNA harbors two trinucleotide repeats in the region encoding its N-terminal domain. Indeed, in a screen of MID1 complex-associated mRNAs we have identified the AR mRNA. In the current study, we examined the role of MID1 in AR signaling and the potential mechanisms by which MID1 may contribute to prostate cancer initiation and/or progression.

## Results

### The AR mRNA associates with the MID1 complex

**Figure 1:**
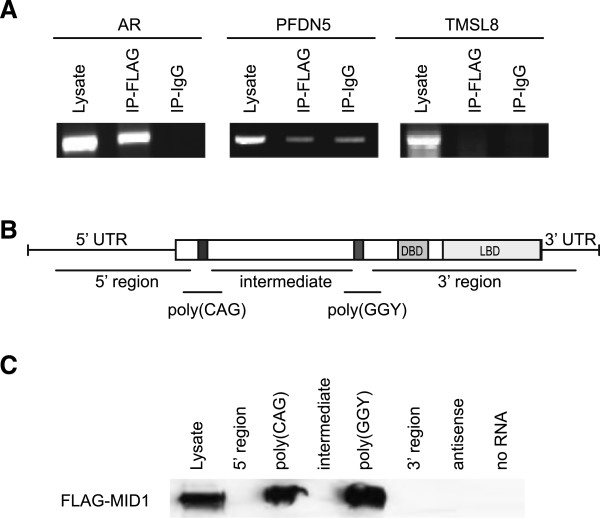
**AR mRNA associates with the MID1 complex. (A)** Agarose gel showing RT-PCR products of AR or of two control genes (prefoldin 5, PFDN5; thymosin like 8, TMSL8). mRNA was isolated either from total cell lysate or after immunoprecipitation of the MID1 complex using lysates from MID1-FLAG overexpressing HeLa cells with an anti-FLAG antibody (IP-FLAG) or with unspecific IgGs (IP-IgG). **(B)** Schematic overview of five different regions on the AR mRNA: 5′ region: nucleotides (nt) 212–1224, poly(CAG): nt 1160–1446, intermediate sequence: nt 1390–2445, poly(GGY): nt 2382–2652, 3′ region: nt 2593–4123. **(C)** Detection of FLAG-MID1 on Western blots analyzing AR mRNA association to MID1. In vitro transcribed and biotin-labeled regions of AR mRNA were added to lysates of HeLa cells overexpressing MID1-FLAG. Streptavidin coated magnetic beads were used to isolate the RNA/protein complexes. Whole lysate served as control for presence of MID1 and antisense RNA or no RNA were used as negative controls.

Previously, we have shown that the MID1-protein complex binds mRNAs via purine-rich RNA motifs, which form stable secondary structures [16,20] including expanded CAG repeats as seen in mutant huntingtin [18]. The two repeat stretches in the 5′ translated end of the AR mRNA, which are purine-rich structures forming stable hairpins [21], are candidates for being recognized by the MID1 complex.To corroborate our initial finding of an association of MID1 with the AR mRNA in a screen originally performed in HeLa cells, we first isolated mRNA from HeLa total cell lysate or from co-immunoprecipitates of the MID1 complex using lysates from MID1-FLAG overexpressing HeLa cells. RT-PCR was performed using primers for the AR and for two control genes (prefolding 5, thymosin-like 8). A specific PCR product was only obtained with the AR-specific primers in the MID1 pull-down sample, indicating that the AR mRNA is bound by the MID1 complex (Figure 1A).

To map putative binding sites of the AR mRNA to the MID1 complex, we performed RNA-protein pull-down assays. As the analysis of respective deletion constructs in cells turned out to be difficult due to altered protein- and RNA-expression/stabilities and activities [22-24], we used similar amounts of five different (Figure 1B) in vitro transcribed, biotinylated AR mRNA fragments, which were incubated with HeLa cell extracts overexpressing FLAG-MID1. RNAs were purified with streptavidin-coated beads and bound proteins were analyzed by Western blot using an anti-FLAG antibody. Control experiments with antisense RNA and without RNA respectively, were performed in parallel (Figure 1C). While there was no binding of MID1 to the 5′, 3′ or the intermediate region, strong interactions of the MID1 protein with the polyCAG repeat and the polyGGY-repeat RNA fragments were detected.

### AR protein levels and activity depend on MID1

**Figure 2:**
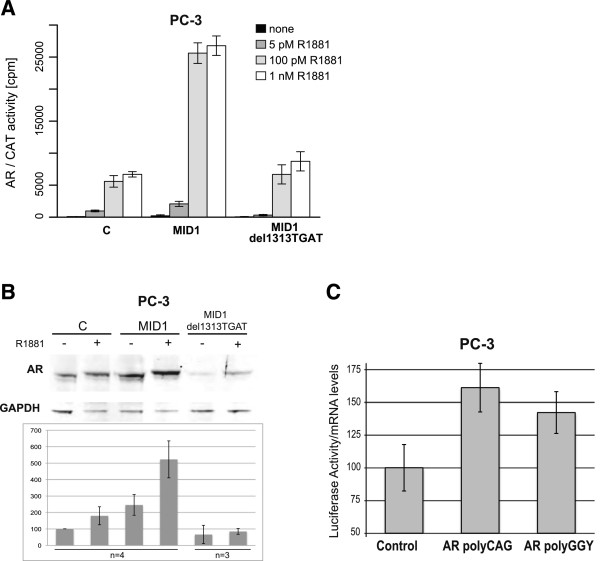
**AR protein levels and activity depending on MID1. (A)** AR-dependent CAT reporter gene assay in presence of different androgen (R1881) concentrations. PC-3 cells were co-transfected with plasmids expressing AR and either wild type MID1, mutated MID1 (del1313TGAT) or empty plasmid as control. CAT activity was measured in counts per minute (cpm) by using the reporter gene construct (ARE)_2_TATA-CAT. Error bars in barplots denote standard deviation (n = 4). **(B)** Western blot analysis detecting AR and GAPDH proteins after co-transfecting PC-3 cells with AR and either MID1, MID1 1313delTGAT or empty vector as control. Densitometric analysis of respective Western blots from several experiments as indicated. **(C)** Luciferase reporter assay showing that the MID1-binding sites on AR-mRNA are responsible for the MID1-dependent translational enhancement of AR: PC-3 cells were transfected with either control reporter vector or a construct with the polyCAG-region or the polyGGY-region of the AR-mRNA cloned into the 3′UTR of the luciferase reporter gene. Luciferase activity was measured and the values relative to the control and normalized to the luciferase-mRNA levels are shown (n = 3).

Next, the question arose, if the MID1 protein is involved in translational control of the AR mRNA. In order to assess the importance of MID1 for AR activity, we performed AR dependent CAT reporter assays. Plasmids incorporating the CAT gene under the control of an AR responsive promoter and wild-type AR were co-expressed with either empty vector, wild-type MID1 or mutant MID1 expression vectors in the AR-negative prostate cancer cell line PC-3. AR activity increased up to 5-fold in the presence of MID1 and androgen (R1881). By contrast, expression of the MID1 mutant del1313TGAC did not enhance AR activity beyond basal levels (Figure 2A).To find out if MID1 influences AR activity via elevated protein levels, we co-transfected the same plasmids used for CAT reporter assay in PC-3 cells and analyzed AR protein levels by Western blot. Increased AR protein levels were detected after co-transfection of wild type MID1 with and without androgen (R1881) addition, but not after co-expression of mutant MID1 (Figure 2B).

Since MID1 overexpression increased the protein level of AR and because MID1 also binds specifically to the purine-rich repeats regions in AR mRNA (Figure 1C), we tested if, when MID1 is co-expressed, these regions could also increase the expression from a luciferase-reporter when introduced into its 3′UTR. Such a functional transferability of MID1-recognition motifs from the coding region into the 3′UTR of a reporter gene has recently been shown [16,17]. There was a clear increase in luciferase activity in PC3 cells when either the polyCAG or the polyGGY regions of AR mRNA were introduced into the 3′UTR of the luciferase reporter gene. To show that these effects were caused by improved translational efficiency and not due to enhanced transcription/mRNA stability, the luciferase activities were normalized to luciferase mRNA levels.

**Figure 3:**
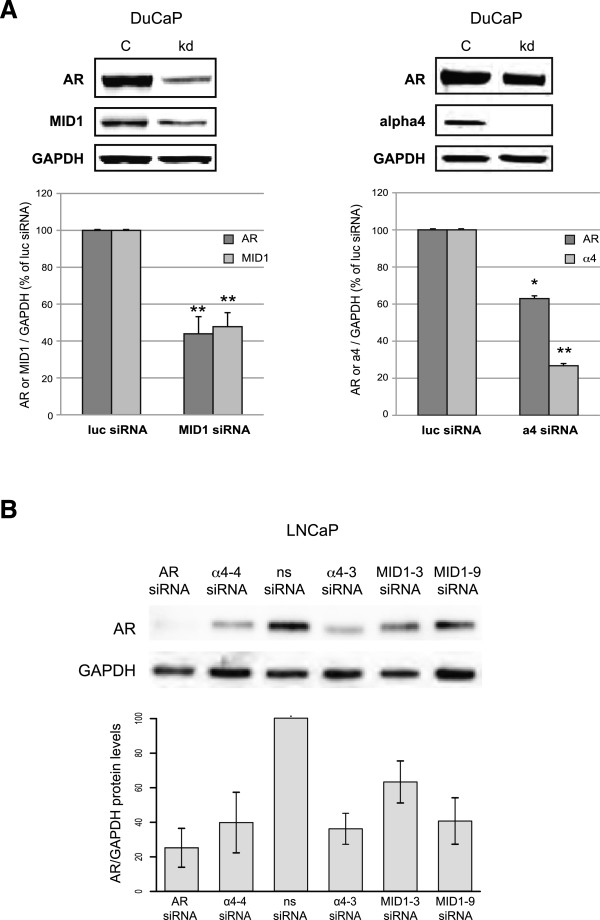
**AR-protein levels in response to MID1 or α4. (A)** Western blot analysis detecting AR and GAPDH proteins in DuCaP cells after knockdown (kd) of MID1 (n = 3) or α4 (n = 2) with siRNAs. Non-silencing siRNA served as control. **(B)** Analogous experiment with LNCaP cells and other siRNAs.

To determine if loss of MID1 or of its direct binding partner α4 has abrogating effects on endogenous AR protein levels, knockdown experiments were performed in DuCaP cells, a prostate carcinoma cell line expressing high levels of endogenous AR. Consistent with our previous results, siRNA oligonucleotides directed against MID1 or α4 dramatically reduced AR protein levels (Figure 3A). The same could be shown in another AR-positive prostate cancer cell line (LNCaP) and with other MID1-specific siRNAs (Figure 3B, Additional file 1: Figure S1A-C). Determination of AR-mRNA levels with qPCR during knockdowns in LNCaP cells showed that AR-mRNA levels are not significantly influenced by MID1/α4 (Additional file 1: Figure S1D). Furthermore, overexpression of MID1 in LNCaP cells does also not lead to significant changes in AR mRNA levels (Additional file 1: Figure S1E), confirming the impact of the MID1 complex on AR translation rather than on RNA-transcription regulation.

### AR mRNA and protein stabilities are not influenced by MID1

**Figure 4:**
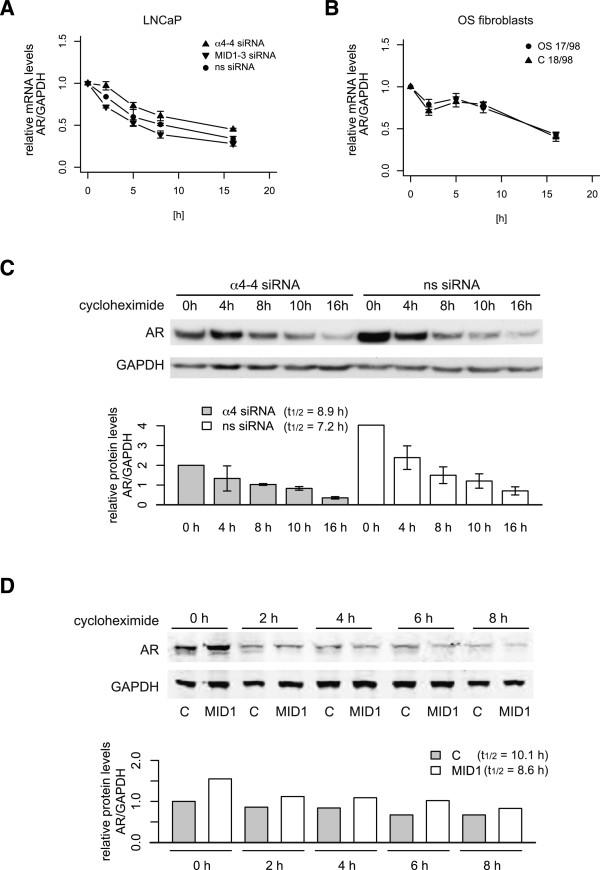
**AR mRNA and protein turnover in response to MID1 or α4. (A)** Real-Time PCR analysis of AR mRNA stability in LNCaP cells treated with non-silencing (ns), α4 or MID1 specific siRNA oligonucleotides. Levels of AR relative to GAPDH mRNA were plotted against time points after ActinomycinD addition. **(B)** Real-Time PCR analysis of AR mRNA stability in fibroblast cells harboring mutant MID1 (OS17/98) and control fibroblasts (C18/98). Levels of AR relative to GAPDH mRNA was plotted against time points indicating ActinomycinD addition (n = 3). **(C)** Western blot analysis detecting AR and GAPDH proteins after knockdown of α4 in LNCaP cells. Samples were taken at indicated time points after cycloheximide addition (n = 3). Densitometric analysis of Western blots is shown (bottom panels). **(D)** Western blot analysis detecting AR and GAPDH proteins after over-expression of FLAG-MID1 (MID1) in PC-3 cells with ectopically expressed AR. Samples were taken at indicated time points after cycloheximide addition. Densitometric analysis of Western blots is shown (bottom panels).

Our data show that MID1/α4 regulate AR protein levels. Despite having ruled out major transcriptional effects this could still be caused by alterations in AR mRNA or protein stability.To analyze AR mRNA stability, knockdown of LNCaP cells with MID1, α4 or non-silencing siRNA oligonucleotides in the presence of the transcription inhibitor actinomycinD showed that the MID1 protein complex had no effects on AR mRNA stability (Figure 4A). Additionally, AR mRNA stability was unaltered in genital skin fibroblast with non-functional MID1, compared to control cells, upon actinomycinD treatment (Figure 4B).To analyze the influence of the MID1 complex on AR protein stability, AR degradation was quantified by Western blot in LNCaP cells that were treated with the translation blocker Cycloheximide after α4 knockdown (Figure 4C) or after MID1 overexpression in PC3 cells with ectopic expression of AR (Figure 4D). Calculation of AR-protein half-life did not show significant differences in AR-protein stability between control and α4 knockdowns and MID1-overexpression did not lead to a stabilization of AR-protein in PC3 cells, again corroborating that the MID1/α4 protein complex regulates AR protein levels neither by alterations in mRNA levels nor of mRNA or protein stability, but by influencing the translation efficiency of AR mRNA.

### Reciprocal feedback regulation of MID1 and AR

**Figure 5:**
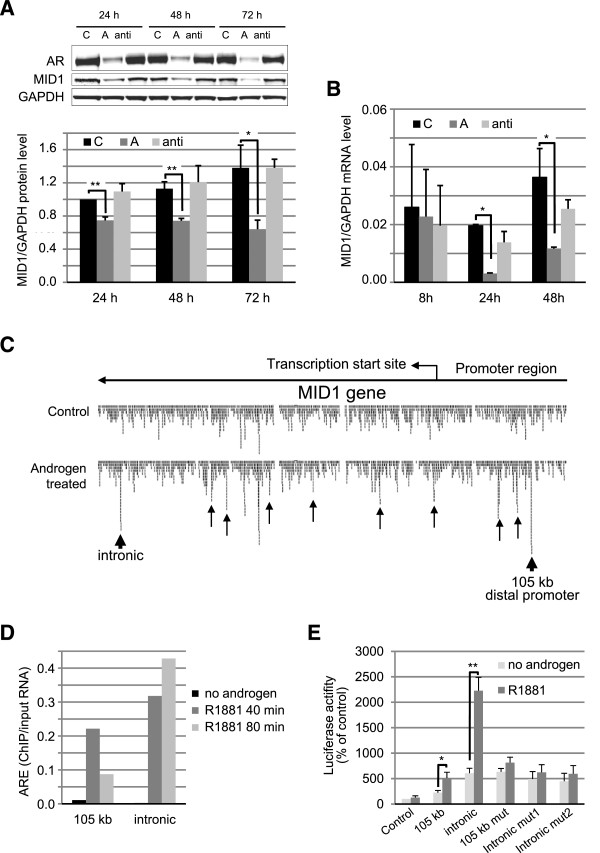
**Reciprocal regulation between MID1 and AR. (A)** MID1 protein levels in response to androgen and anti-androgen treatment. Western blot analysis (upper part) detecting MID1, AR and GAPDH after treatment with the synthetic androgen R1881 or the androgen antagonist bicalutamide at indicated time points. Lower part: bar-graph of densitometric analysis of respective Western blots from 3 experiments. **(B)** MID1 mRNA levels in response to androgen and anti-androgen treatment. Real-Time PCR analysis of MID1 relative to GAPDH mRNA levels in DuCaP cells after treatment with R1881 or bicalutamide. **(C)** The MID1 gene comprises androgen response elements (AREs) inside its promoter and in intronic regions. Graphical demonstration of AR binding sites determined by Chromatin Immunoprecipitation (ChIP) coupled with sequencing in the AR positive DuCaP cell line. Differential peaks between control and androgen treated panels indicate AR binding to these regions. Arrows show AR binding sites, circles show binding sites selected for functional validation. **(D)** Confirmation of selected AR binding sites in an independent ChIP sample set. Selected AR binding sites (intronic and 105 kb distal promoter sites) were amplified with specific primers and the enrichment of AR bound fragments was determined by Real-Time PCR in ChIP samples in response to androgen (R1881) or vehicle treatment. **(E)** Functionality of AREs in the MID1 gene. AR-dependent luciferase reporter assays in AR-positive DuCaP cells were performed to assess the function of the 105 kb distal promoter and the intronic AR binding sites. Reporter vectors carrying either binding site DNA fragments or the binding site sequences with mutations introduced into the predicted AREs were transfected into DuCaP cells and cells were treated with R1881 or vehicle control for 24 h. (n = 3).

Previous microarray data performed by our group showed MID1 to be negatively regulated by androgens in various AR positive prostate cancer cell lines (unpublished data). To specify these findings, MID1 protein levels were measured at several time points in the AR-positive prostate cancer cell line DuCaP after treatment with synthetic androgen R1881. MID1 protein levels clearly decreased after 24 h, 48 h, and 72 h (Figure 5A). Similarly, AR protein was downregulated upon androgen treatment, which is a characteristic property of DuCaP cells that does not impede induction of AR regulated genes [25]. Next, we tested whether androgen treatment of DuCaP cells also influences MID1 mRNA levels. Consistent with our previous microarray data, MID1 mRNA levels were significantly decreased after 24 h and 48 h (Figure 5B). Considering these results, we hypothesized that MID1 could be feedback regulated by the AR.To address the potential inhibitory function of the AR on MID1 expression, we performed chromatin immunoprecipitations (ChIP) sequencing using DuCaP cell lysates and found several AR binding sites (AREs) in both the MID1 promoter region and within intron regions (Figure 5C). Two of these MID1-AREs, one in the distal promoter region and one in an intronic region of MID1, were selected for further investigation. Independent ChIP experiments performed upon androgen stimulation resulted in the enrichment of both AR binding sites in the ChIP samples, precipitated using an AR antibody at different time points (Figure 5D).

To confirm our findings, luciferase reporter vectors were constructed including the sequences of these two selected binding sites. Consensus AREs in the two binding sites were indentified *in silico* using the online web-based tool Math Inspector. One consensus sequence in the 105 kb distal promoter and two consensus sequences in the intronic region were determined. These consensus sites were mutated using site-directed mutagenesis. Reporter gene activities of these constructs were tested in DuCaP cells with or without androgen stimulation. Introduction of the putative AR binding sites into the reporter gene promoter slightly increased basal luciferase activities and significantly increased androgen-induced reporter gene activities whereas mutation of MID1-AREs abrogated the effect of androgens (Figure 5E), thus confirming the functionality of AR-binding on these two selected regions. Taken together, our results suggest that MID1 is an AR target gene and is negatively regulated by androgens. Given the critical role of AR activation in prostate cancer initiation and progression, negative feedback regulation of MID1 by the AR may be an important and effective way in order to strictly control AR signaling.

### MID1 is highly expressed in prostate cancer tissues

**Figure 6:**
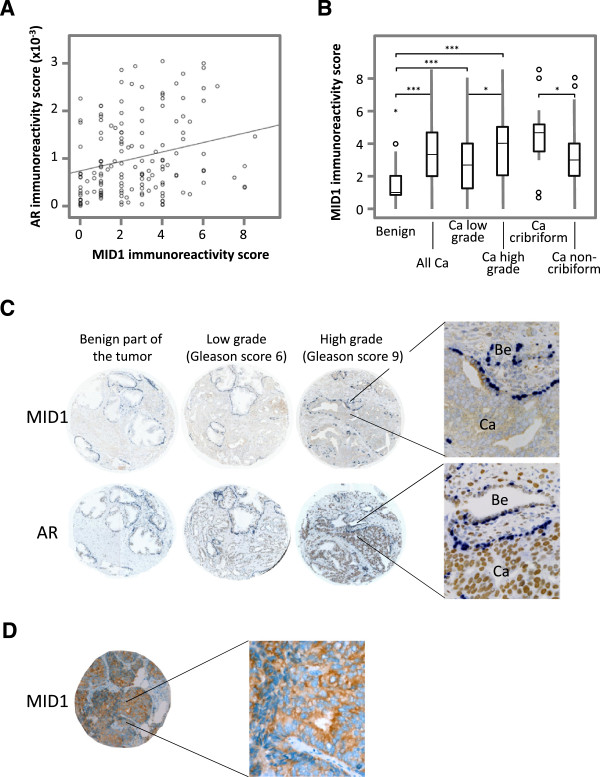
**A prostate cancer tissue microarray containing tissue samples from 94 patients with primary prostate cancer (3 cores of cancer and 1 core of benign for each case) were analyzed for MID1 and AR by IHC. (A)** Correlation of AR and MID1 immunoreactivity is shown. R = 0.246 according to Pearson and R = 0.263 according to Spearmen correlation analysis being both significant at p = 0.01 level. **(B)** MID1 expression pattern in different histological and pathological cancer categories. 79 benign and 88 cancer samples were evaluated and included in the analysis. One-way Anova with Posthoc Bonferroni or Duncan was used for categories containing more than 2 groups and Mann Whitney *U* test for categories containing only 2 groups (benign and cancer). *p < 0.05, **p < 0.01,***p < 0.001. **(C)** Representative images of AR and MID1 immunohistochemistry in low and high grade cancer. Consecutive sections were double stained for AR or MID1, respectively (brown), and the basal cell marker P63 (dark blue). **(D)** Analogous images of tissue sections from two metastases.

To further address the potential role of MID1 in prostate cancer, we evaluated MID1 and AR expression levels by immunohistochemistry (IHC) in prostate cancer specimens. A statistically significant positive correlation between MID1 and AR expression levels was found (Figure 6A) (R = 0.246 and 0.263 according to the Pearson and Spearman correlation analysis, respectively, p = 0.01). Although the expression pattern of MID1 was heterogeneous, some trends were obvious: in the non-malignant benign parts of the specimens MID1 expression was mainly stromal, consistent with minimal AR expression in these cells (Figure 6C, first panel). MID1 expression was enhanced in tumor areas in correlation with increasing Gleason grade of the tumors (Figure 6B and C second panel compared to third panel). MID1 expression level was significantly increased not only in cancer compared to benign but also increased in high compared to low Gleason score tumors (Figure 6B). Notably, high MID1 expression was especially correlated with a histological tumor pattern referred to as cribriform. When the cancer samples were categorized to cribriform and non-cribriform sets, MID1 expression level was significantly higher in the cribriform sample set (Figure 6B). Furthermore, highest expressions levels in cribriform pattern tumors was confirmed with lymph node metastases (Figure 6D), although the number of lymph node samples available for the study was too small for a statistical analysis. Taken together, our results suggest modified MID1 expression in prostate cancer along with high AR expression levels, reflecting one mechanism that contributes to prostate cancer progression.

## Discussion

In this study, we identified the MID1 complex as a physiologically important mediator of AR signaling, which acts through the newly identified mechanism of enhancing AR mRNA translation. Many studies have shown that the AR pathway is crucial for prostate cancer progression and that this pathway maintains its role even after androgen depletion and development of resistance to therapy. Several mechanisms were proposed to explain the role of AR in this transition to therapy resistance. Increased expression of AR was reported for a vast majority of prostate cancer tissue samples, achieved through mechanisms such as AR gene amplification, which was found approximately in one third of patient samples [26], or post-transcriptional regulation.

Post-transcriptional regulation of AR mRNA is a mechanism not well described in the literature. The AR mRNA 3′UTR has UC rich regions, which are targeted by RNA binding proteins (RBPs). HuR and poly-C binding protein 2 (PCBP2) were reported to bind the 3′UTR of AR and regulate it posttranscriptionally [27,28]. In addition, poly-C binding protein 1 (PCBP1) was identified as a negative regulator of AR mRNA translation in a 3′UTR-independent manner [29]. Of note, the AR harbors several repeat regions in its 5′translated region, which are principally also good candidates for the binding of RBPs [30]. However, until now no RBPs have been reported to bind these repeats. To our knowledge, we have here identified the MID1/α4 complex as the first positive regulator of AR translation, associating with the AR mRNA through these repeat regions. In a recent publication, it was shown that the MID1 complex associates with purine-rich motifs, named MID1-association sequences (MIDAS). In the presence of the MID1 complex, MIDAS-containing mRNAs are translated more efficiently [16]. Furthermore, in a second paper we show that MID1 enhances the translation of mutant huntingtin mediated by its binding to expanded CAG repeat stretches [18]. While wild type huntingtin mRNA that harbors 20 consecutive CAGs shows only weak binding to the MID1 complex, mutant huntingtin with 51 CAGs shows strong binding resulting in mTOR dependent enhanced translation [18]. The CAG repeat stretch present in the AR mRNA (23 CAGs + 6 adjacent CAGs) is shorter than in mutant huntingtin, but the AR mRNA additionally harbors a repeat with 23 consecutive GGYs that supports binding to the MID1 complex and would finally lead to translation enhancement of AR. Here, we show that the MID1 complex binds to the polyCAG and polyGGY repeat regions of AR mRNA and that these repeats when introduced into the 3′UTR of a reporter gene, can increase the translational efficiency from the reporter. Furthermore, enforced expression of MID1 in PC-3 and DuCaP cells dramatically enhances AR protein levels. Interestingly, this correlates with an even higher increase in AR transcriptional activity what could be explained by additional indirect effects of the MID1 over-expression, like altered phosphorylation patterns of AR caused by the influences of MID1 on kinases as AKT and mTOR as well as on the phosphatase PP2a [15,16,31]. Additionally, siRNA knockdown of endogenous MID1/alpha4 lowers AR expression to almost the same extent as an siRNA knockdown of AR itself. Notably, neither AR mRNA levels nor mRNA/protein stability were influenced by MID1 to a similar extent, confirming MID1’s role as a positive translational regulator.

**Figure 7:**
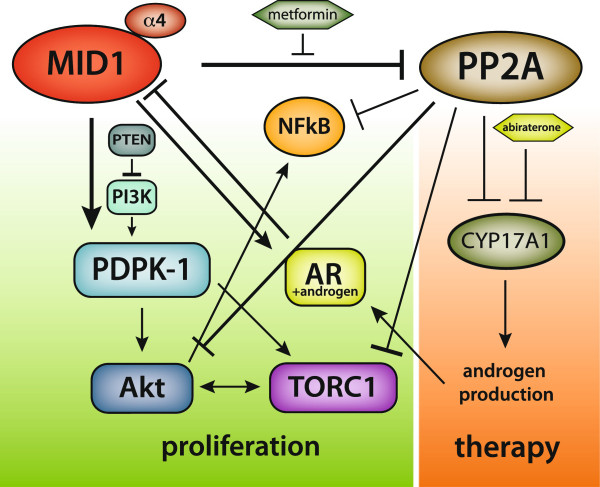
**MID1 is an upstream modulator of proliferation.** MID1 enhances protein levels of PDPK-1 and AR, resulting in promotion of PI3K and AR signaling pathways. Moreover, PP2A, which inhibits Akt and mTOR signaling as well as androgen hormone production, similarly as the approved anti-prostate cancer drug abiraterone, is targeted for degradation by MID1, a process that can be inhibited by metformin. Moreover, MID1 can activate the survival factor NFκB via inhibition of PP2A. Thus, MID1 drives proliferation by enhancing respective proteins and hormones, however, there is a negative feedback loop between MID1 and AR: MID1 enhances AR post-transcriptionally, which then in the presence of androgens translocates to the nucleus and, acting as transcription factor, negatively regulates MID1 gene expression. Thus, androgen-ablation therapy could lead to an increase in MID1, which then promotes proliferation and paves the way for PCa progression.

Mechanisms that regulate AR levels are intensively studied due to the crucial role of AR in prostate development and maintenance. One mechanism reported in the literature is auto-regulation of AR by androgens. In this setting, AR down-regulates its own transcription by binding putative AREs inside the AR gene when it is bound to androgen [32-35]. In a recent report, the responsible ARE in the AR gene was located to a highly conserved site in the second intron. In response to androgen, liganded AR recruited lysine-specific demethylase 1 (LSD1) to this site to reorganize chromatin structure, leading to activation of a negative feedback to regulate AR activity by directly inhibiting AR transcription [36]. In our study, we propose a novel regulatory loop between MID1 and AR (Figure 7). While MID1 acts as a translational inducer of AR protein, AR in turn decreases MID1 levels in response to androgen stimulation. Therefore, AR maintains its own levels in equilibrium through MID1. A similar negative regulation between PMEPA1 and AR was proposed by Li et al. via a proteasome-dependent mechanism [35].

We identified several AR binding sites in the MID1 gene by ChIP-Seq analysis. Consistent with current understanding [26], AREs of the MID1 gene are located either within distal promoter regions or in intronic regions. Although we confirmed the principal functionality, namely triggering androgen-modulation of transcription, of these putative AREs within selected AR binding sites, the negative effect on gene expression, as observed for MID1, could not be shown with the reporter assays used. This indicates the involvement of sequence elements other than AREs or maybe combinations of different AREs that are necessary to assemble an inhibitory AR complex and provide negative regulation. Commonly, transcription factor binding sites for SP1, AP1, GATA2, Oct1, ETS, FOXA1 are enriched around AREs to fine tune AR regulated transcription [26,37,38]. Further studies are obligatory to propose a model in which AR decreases transcription and which other factors are involved in the negative regulation of MID1 with androgen. One possible candidate responsible for AR/androgen-dependent repression of MID1 could be the known AR-co-repressor “small heterodimer partner (SHP)”. Interestingly SHP is involved in several of the pathways that could be correlated with the MIDAS-dependent translational regulations of MID1, such as lipid metabolism, glucose homeostasis, proliferation and migration/angiogenesis [16,39].

The regulatory impact of MID1 on AR and its possible implications in PCa suggested a role of MID1 expression in development and progression of PCa. Our IHC results with samples of prostate cancer tissues revealed that MID1 is mainly stromal in benign tissue areas. Interestingly, in cancerous tissue the MID1 expression shifted to the epithelial compartment and was excessive in higher-grade tumors and metastatic lesions of prostate cancer. This shift may be due to alterations of the regulatory loop between AR and MID1 which could be correlated with the reported stage-dependent reductions in androgen levels in PCa tissues [40] and in the blood circulation of PCa patients [41,42]. It is a well-established fact that during androgen ablation therapy, tumors adapt to low androgen environment by changing AR mediated growth response. MID1 expression is reduced in case of normal androgen levels, whereas androgen ablation reliefs the negative feedback on MID1 and leads to increased MID1 levels, which in turn could enhance AR levels via a translational mechanism. This scenario would suggest a role of disrupted MID1-AR equilibrium in the progression of prostate cancer to an androgen ablation therapy resistant stage.

In addition, MID1 seems to positively modulate other important oncogenic players, like PDPK1, which is a crucial kinase in the PI3K pathway and a novel promising target for anticancer drugs – its mRNA harbors a MIDAS motif in its 3′UTR, which triggers MID1-dependent enhanced translation [16]. Notably, PDPK1 is oncogenic through stimulation of EMT and cell migration, and upregulation of the pro-metastatic metalloproteinase MMP14 [7,43,44]. The latter also harbours a MIDAS motif, which suggests a further MID1-driven up-regulation. Indeed, elevated levels of MMP14 have also been observed during PCa progression into castration resistance [45]. The PI3K/PDPK1/AKT pathway is crucial for prostate cancer progression and a recent study showed a reciprocal regulation between the AR and the AKT pathway. This finding suggests that the failure of androgen ablation therapy might be partially due to the induction of the PI3K/PDPK1/AKT survival pathway in the absence of androgens [3]. Increased MID1 levels through androgen ablation would lead to the enhanced translation of PDPK1 and subsequent activation of the AKT signaling cascade. Interestingly, pharmacologic inhibition of AKT and AR hamper androgen-independent growth of LNCaP cells. Taken together, the MID1 complex may be a promising therapeutic target in castration resistant prostate cancer, because in the absence of androgens it can modulate several major oncogenic players.

An interesting compound in this context is the anti-diabetic drug metformin that was identified as a disruptor of the MID1 protein complex [46]. Very recently, we could also show that the anti-tumor effect of metformin in prostate cancer cells is in part caused by its disruptive effect on the MID1 protein complex and the subsequent downregulation of the AR protein [47]. This mechanism is fully in line with the regulatory role of MID1 on AR protein levels presented here. Furthermore, the observed accumulation of PP2A caused by the disruption of the MID1 complex through metformin [46] could have a repressive effect on androgen synthesis, as PP2A is a strong negative regulator of cytochrome P450c17 and 17,20 lyase activities, which are both crucial for androgen synthesis. In view of the findings that adrenal and local androgen synthesis can drive AR in castration resistant prostate cancer this would even enhance the inhibitory effect of metformin on tumor progression [48]. Interestingly, cytochrome P450c17 (CYP17A1) activity is also targeted by abiraterone [12], an approved drug used for the treatment of castration-resistant PCa (Figure 7).

Interestingly, the benefits seen with metformin in treatments of polycystic ovary syndrome (PCOS), a condition partially caused by increased androgen signalling [44], could also be explained by metformin’s inhibitory effect on MID1 that results in reduction of androgen receptor translation and concomitantly in reduction of androgen synthesis via increase of PP2A. In this context it should be mentioned that such a bifurcated inhibition of androgen signalling by disruption of MID1’s function might also underly the phenotypical similarities Opitz patients share with patients suffering from partial androgen insensitivity syndrome, namely hypospadias.

Finally, it was recently reported that metformin negatively regulates signalling via NFκB [43] which is a pivotal player in prostate cancer progression. This effect of metformin would also be in line with its inhibitory impact on the MID1 complex [46], which has recently been shown to promote inflammation via NFκB activation [8].

The awareness of MID1 as an essential regulator of a pool of specific mRNAs involved in cancer development and progression is a novel concept. Three translational studies support such a role of the MID1/α4/PP2A complex: (i) a study on colorectal cancer, pointing towards MID1 as a metastatic gene and marker for poor survival [49], (ii) a study, which demonstrated that α4, the direct binding partner of MID1, is expressed universally in advanced lung adenocarcinomas and that its overexpression is significantly related to outcome [50], and (iii) a study, which shows that the expression and activity of PP2A is down-regulated in castration resistant prostate cancer cells [51]. Finally, our present findings and recent reports regarding the crosstalk between MID1/α4 and Akt/mTOR, [15,16,46], two master regulators of essential pathways in proliferation, development, energy balance and immune responses, as well as MID1’s roles in NFκB activation [8] and promotion of Hedgehog signaling [13] stress the multifaceted nature and therapeutic potential of the MID1 complex.

## Conclusion

The ubiquitin ligase MID1, which is over-expressed in prostate cancer tissue in a stage-dependent manner, enhances androgen receptor protein levels. This upregulation occurs at the translational level and is reciprocally controlled by an androgen-dependent repression of MID1 expression by androgen receptor at the transcriptional level. The disruption of this feedback-loop by androgen withdrawal could play a role in the development of castration resistant prostate cancer.

## Methods

### Cell culture procedures and antibodies

DuCaP and PC-3 cell lines were cultured in RPMI 1640 medium supplemented with 5-10% FBS 1% Glutamax and 1% penicillin/streptomycin (P/S) LNCaP cells were cultured in MCDB-131 medium supplemented with 10% FBS, 10 mM Hepes, 0.45% glucose, 1% sodium-pyruvate and 1% P/S. Fibroblast cells harbouring mutant MID1 (OS17/98) and control fibroblasts (C18/98) were established from a patient and a healthy control subject, respectively, after written informed consent. Cells were maintained at 37°C and 5% CO_2_. The concentration of reagents used for in vitro experiments was: synthetic androgen R1881: 1 nM, anti-androgen Bicalutamide: 2.5 μM, Cycloheximide: 80 μg/ml and ActinomycinD: 5 g/ml. For transfection of expression plasmids Attractene (Qiagen) was used for PC-3 cells, Lipofectamine (Invitrogen) for LNCaP and Nanofectin (PAA) for DuCaP and PC-3 cells. Antibodies for Western blot, immunoprecipitation and ChIP were obtained from Biogenex (anti-AR, mAb), Chemicon (anti-GAPDH, mAb), Cell Signaling (3202, anti-AR, pAb) and from Millipore (UB 06–680, anti-AR, pAb), Stratagene (anti-FLAG) Abcam (anti-α4, pAb) and from Atlas Antibodies (anti-MID1, pAb).

### Constructs

Several MID1 variants (wild-type MID1, MID1 Ala130Thr, MID1 del1313TGAT and MID1 IVS8 open reading frames) were inserted into the multiple cloning site of the pCMV-Tag2C vector (Clontech) using EcoRI and HindIII. For investigating the influence of the polyQ and polyG regions of AR mRNA on mRNA translation these region were PCR amplified and inserted into the 3′ UTR of the luciferase reporter gene in a pGL3 luciferase reporter plamid (Promega).

For CAT reporter gene assays AR expression vector AR-pSG5, reporter plasmid (ARE)2-TATA-CAT and fill-up plasmid pHRL-Null were used, for Dual-Luciferase Reporter Assays the reporter plasmid was replaced by plasmids constructs of the pGL3 luciferase promoter vector (Promega) and the renilla firefly expression plasmids pGL4.70 or pGL4.73 were added for normalizing transfection efficiency. Luciferase reporter plasmids containing MID1 105 kb promoter and intronic AR binding sites were constructed by inserting the PCR-amplified AR binding site fragments into the pGL3-promoter vector. The consensuses AREs in these AR binding fragments were mutated using the QuickChange II Site-directed Mutagenesis kit (Agilent).

### Primers

Primers used for amplification of AR binding site fragments and primers for site-directed mutagenesis

105 kb promoter binding site:

F: 5′ AGGAGTGACAGCAATGATTTCAGGG′3,

R: 5′ AGTGCACACATGTGCCAGACCC′3;

ARE Mutagenesis:

F: 5′CCTTAAGTATGGAGAT ***G*** AGGTT ***AGG*** CCTTGATGTTGCCCTAGGC′3,

R: 5′ GCCTAGGGCAACATCAAGG ***CCT*** AACCT ***C*** ATCTCCATACTTAAGG′3.

Intronic binding site:

F: 5′TCTCCAGCCTCCTGGCTCACCTA′3,

R: 5′ AGTTGACAAAGCCAGGGTGCCC′3;

ARE1 mutatenesis:

F: 5′TCTCTGGGCTTTTGCC ***G*** ATGCT ***AGG*** CCTTCTGCATGGA′3,

5′TCCATGCAGAAGG ***CCT*** AGCAT ***C*** GGCAAAAGCCCAGAGA′3,

ARE2mutagenesis:

F: 5′TTCTGCATGGAA ***G*** ACTGT ***AGG*** CTCACATCCCCTGCTCC′3

R: 5′ GGAGCAGGGGATGTGAG ***CCT*** ACAGT ***C*** TTCCATGCAGAA′3

### Immunoprecipitation

4×10^6^ HeLa cells overexpressing FLAG-MID1 were lysed in TKM buffer (20 mM Tris–HCl, pH 7.5, 150 mM KCl, 5 mM MgCl_2_), supplemented with proteinase inhibitors and 1% NP40, incubated for 15 min on ice and passed 6 times through a 27¾ gauge needle and centrifuged for 15 min at 12000 × g at 4°C. 4 mg of cytosolic extract were precleared with 50 μl of protein-A/G agarose (Roche) and 10 μg of mouse IgG for 2 h at 4°C on a rocking platform. Beads were pelleted and discarded. The supernatant was immunoprecipitated with 50 μl of anti-FLAG M2 affinity gel (Sigma-Aldrich) over night. As control, the IP was carried out with mouse IgG. Agarose beads were pelleted and washed 3 times with 500 μl TKM buffer supplemented with 0.2% NP40 for 10 min at 4°C. Bound proteins were finally eluted for 1 h with 30 μl of 3× FLAG peptide (5 mg/ml), diluted with 200 μl TKM-buffer and 1 μl RNasin. 50 μl were directly analyzed by Western blotting. From the remaining elution fraction bound RNA was isolated by phenol/chloroform extraction followed by EtOH precipitation. cDNA was synthesized using random primers and subsequently used for RT-PCR with AR (F: CTTCTGCACGAGACTTTGAG, R: CTGAAGGAGTTGCATGGTG), PFDN5 and TMSL8 (negative control) specific primers.

### Real-time PCR

To quantify mRNA levels, a one-step real-time PCR method was used, which combines reverse transcription with real-time PCR (Qiagen). Taqman probe-set containing both gene-specific primers and probe for MID1 and housekeeping gene GAPDH were used for amplification and detection on an ABI 7500 Fast PCR machine (Applied Biosystem).

### RNA-protein binding assay

Amplification of different AR mRNA fragments: All forward primers contained the T7 promoter sequence (5′-CCAAGCTTCTAATACGACTCACTATAGGGAGA-3′) to allow subsequent in vitro transcription of the PCR product. PCRs were performed under standard conditions using pSG5-AR plasmid as template and gene region-specific primers for the PCR reactions. Amplified transcripts were in vitro transcribed with the RiboMAXTM Large scale RNA production system-T7 (Promega), following the manufacturer’s instructions with some modifications. Briefly, 4 μg of purified PCR product was transcribed for 4 h at 37°C in the following reaction mixture: 4 μl T7 transcription buffer, 6 μl rNTPs (25 mM rATP, rGTP, rCTP, 1,6 mM biotin-rUTP, 2,5 mM UTP), 4 μg PCR template, 2 μl RNA polymerase enzyme mix, DEPC-H_2_O up to 20 μl. Transcribed RNA was purified and products were kept in nuclease free TE.

RNA-PROTEIN binding assay: 2 μg of biotinylated and purified RNA were incubated with 150 μg of cytosolic protein extract from HeLa cells overexpressing FLAG-MID1 in 450 μl of TKM buffer for 1 hour at 4°C. Subsequently, the mixture was incubated for 2 h at 4°C with 40 μl of 50% slurry of M280 streptavidin coated magnetic beads. Beads were washed 3 times with TKM buffer for 10 min at 4°C. Bound proteins were eluted by boiling the beads in magic mix (2×: 48% urea, 15 mM Tris–HCl pH 7, 8.7% glycerol, 1% SDS, 143 mM mercaptoethanol) for 10 min at 95°C and analyzed on Western blots using the respective antibodies.

### Luciferase reporter gene assays

Reporter gene assays were accomplished according to the Promega Dual Luciferase Reporter gene assay protocol and luciferase activity was measured in a Chameleon V plate reader (Hidex). Cells were transfected with 0.25 μg DNA/well in 24-well format and 0.08 μg DNA/well in 96-well format respectively and kept in medium with 10% charcoal stripped FCS for 24 hours after transfection and treated with 1 nM R1881 for additional 24 hours before luciferase activity was measured. For polyQ/polyG Luciferase Reporter gene assays 0.15 μg reporter vector DNA/well were used to transfect 11,000 PC-3 cells/well in a 96-well format. Cells were kept in RPMI medium with 5% charcoal stripped FCS for 24 hours after transfection and treated with 1 nM R1881 for additional 24 hours.

The polyQ and polyG regions of AR mRNA inserted into the 3′ UTR of the luciferase reporter gene were: gb/M23263.1 nt633-nt816 (polyQ) and gb/HM010955.1 nt2448-nt2616 (polyG).

### siRNA transfection

Cells were transfected with 20 to 40 nM final concentration of MID1, α4, AR or control siRNA oligonucleotides using Lipofectamin 2000 or Nanofectin. AR siRNA: 5′-GAAAGCUCCUCGGUAGGUC-3′; α4-4 siRNA: 5′-UUGAGAUGCCAUAGCAACGAG-3′; MID1-3 siRNA: 5′-GUGUGAUACUAGGAUGCGG-3′; ns siRNA: 5′-AAGAGGCUUGCACAGUGCA-3′ (Dharmacon or MWG). After 48 h of incubation the medium was replaced and after 72–120 h cells were harvested and analyzed by Western blotting or real-time PCR.

### Immunohistochemistry

Paraffin-embedded primary tumor and lymph-node metastasis specimens were obtained from previously untreated patients who had undergone radical prostatectomy at the Department of Urology, Innsbruck Medical University. Use of patients’ samples was approved by the ethics committee at the Innsbruck Medical University and written informed consent was obtained from all patients. Tissue microarrays (TMAs) were produced from the tumor specimens using a manual tissue arrayer. Immunohistochemistry (IHC) analysis was performed with 5 μm TMA sections employing the Ventana Discovery - XT staining automat (Roche, Mannheim, Germany). Standard CC1 (Tris-borate pH = 7,8, 48 min at 98°C) pre-treatment for antigen retrieval was followed by incubation for 1 h with a polyclonal anti-MID1 antibody or a monoclonal anti-AR antibody diluted in antibody diluent, followed by secondary universal antibody solution for 30 min, staining with DAP map kit and counter staining for 4 min with haematoxylin II bluing reagent (all IHC reagents from Roche). Specificity of staining was controlled by including a control antibody (DAKO). Consecutive sections were HE stained or stained for the tumor marker AMACR and the non-malignant benign gland specific marker P63 for correct assignment of non-malignant and cancer areas. MID1 staining was evaluated and scored by a pathologist (G.S) according to the quick score method and AR staining was evaluated by an automated IHC image acquisition and analysis system (HistoQuest, TissueGnostics). This analysis system uses nuclear counterstaining as a reference and calculates intensity and percentage of specific staining of the target antigen as described previously [52].

### Chromatin-immunoprecipitation (ChIP)-coupled deep sequencing and PCR

ChIP was performed as previously described [53]. In brief, DUCaP cells were treated with 1 nM R1881 or control vehicle equivalent for 1 h before formaldehyde crosslinking, DNA sonication and immunoprecipitation with 2 combined AR polyclonal antibodies. Normal rabbit IgG was used as control. The antibody-protein-DNA complex was pulled down, digested and reverse cross-linked. DNA was purified with CHIP DNA kit (Zymo Research, Irvine, CA).

AR antibody precipitated DNA samples were used either for deep sequencing or PCR amplification. Preparation of the libraries and sequencing were performed using the Solexa sequencing platform (Illumina) following the manufacturer’s instructions. DNAs were also subjected to amplification by primers listed above.

### Bioinformatic analyses

Sequencing data were analyzed as previously described [53]. Illumina analysis pipeline software was applied and bases were called by Bustard and aligned to the unmasked human reference genome (NCBI v36, hg18) using BOWTIE [54]. MACS tool was used to identify AR-enriched regions in a genome-wide manner [55]. Read-data was visualized using a local installation of the Generic Genome Browser (http://promotion.molgen.mpg.de/gb2/gbrowse/Human/).

### Statistical analysis

Student *t*-test was used to compare two groups. For statistical analysis of AR-IHC, samples were categorized based on histological and pathological parameters. One-way Anova with Posthoc Benforoni or Duncan for categories containing more than 2 groups (benign, cribriform and non-cribriform; benign, Gleason score ≤6 and Gleason score >6) and Mann Whitney *U* test for categories containing 2 groups (benign and cancer) were used for statistical analysis. Pearson and Spearmen correlation analysis were used for studying MID1 and AR correlation. Statistical significance is indicated as *p < 0.05, **p < 0.01,***p < 0.001, Error bars denote standard deviations except for box blots where they denote minima and maxima.

## Competing interests

The authors state that they have no competing interests.

## Authors’ contributions

RS, HK and SS designed and supervised the study; AK performed knockdowns, reporter gene assays, overexpressions and protein stability experiments and statistics. ÜD performed knockdowns, reporter gene assays and statistics. ÜD and HB performed ChIP experiments. EK, JA, SK and BA performed the Co-IP, RNA-protein binding and mRNA level/stability experiments. AIK and CA provided expert guidance in cell culture methods. HB AW and MRS performed ChIP-Seq analysis. GS performed IHC analysis. AK prepared the manuscript, AK and ÜD prepared figures, ÜD, SS, RS and HK reviewed and revised the manuscript. All authors approved the final manuscript.

## Supplementary Material

Additional file 1: Figure S1 **(A)** Westernblot as knockdown control for MID1-3 and MID1-9 siRNAs shown by reduction of FLAG-tagged MID1 overexpressed in LNCaP cells, **(B,C)** Westernblots as knockdown-controls for the α 4-3 and α 4-4 siRNAs using an antibody against endogenous α 4 in LNCaP cells **(D)** Real-Time PCR analysis of AR mRNA levels in LNCaP cells after knockdown of MID1 or α 4 relative to the non-silencing controls. **(E)** Real-Time PCR analysis of AR mRNA levels in LNCaP cells after over-expression of MID1 or α 4 relative to the control (empty vector). Click here for file
