# Supplementary material for: A hormone-dependent feedback-loop controls androgen receptor levels by limiting MID1, a novel translation enhancer and promoter of oncogenic signaling
Source: Mol Cancer. 2014 Jun 9;13:146. doi: 10.1186/1476-4598-13-146 (PMC4074869; doi:10.1186/1476-4598-13-146)
Supplement: Additional file 1: Figure S1 — (A) Westernblot as knockdown control for MID1-3 and MID1-9 siRNAs shown by reduction of FLAG-tagged MID1 overexpressed in LNCaP cells, (B,C) Westernblots as knockdown-controls for the α 4-3 and α 4-4 siRNAs using an antibody against endogenous α 4 in LNCaP cells (D) Real-Time PCR analysis of AR mRNA levels in LNCaP cells after knockdown of MID1 or α 4 relative to the non-silencing controls. (E) Real-Time PCR analysis of AR mRNA levels in LNCaP cells after over-expression of MID1 or α 4 relative to the control (empty vector). [file 1476-4598-13-146-S1.pdf]

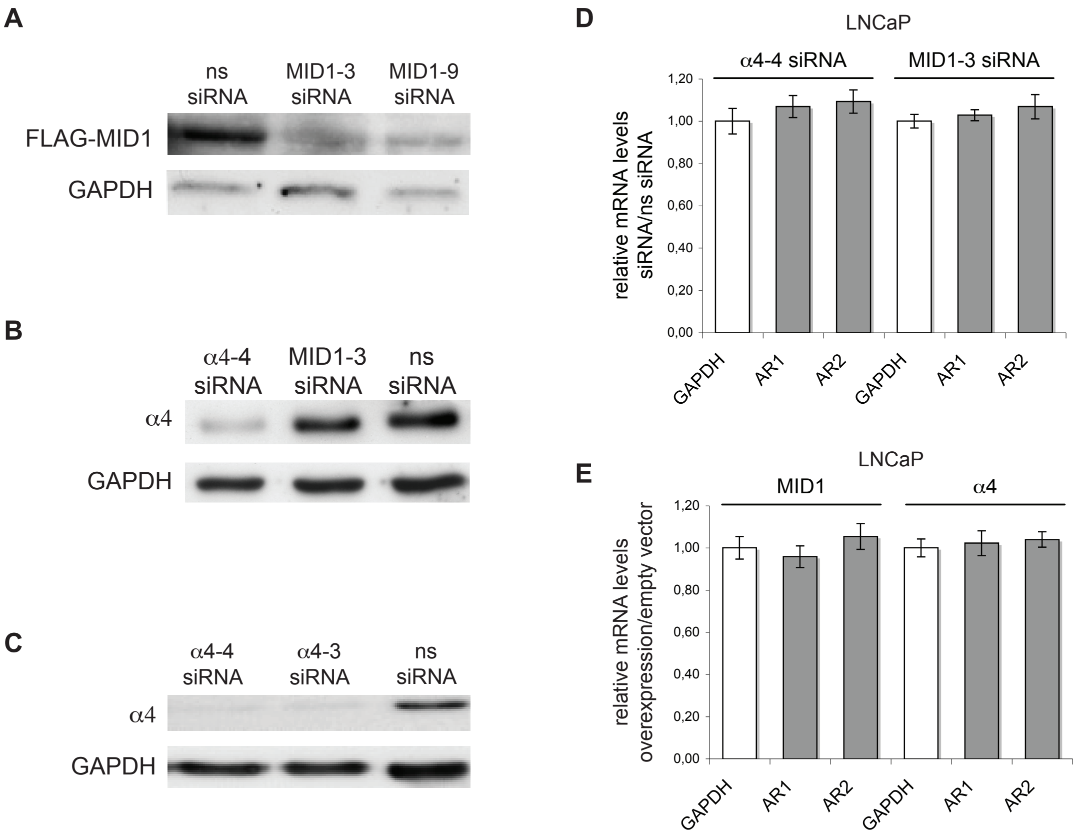
A
ns siRNA
MID1-3 siRNA
MID1-9 siRNA
FLAG-MID1
GAPDH
B
α4-4 siRNA
MID1-3 siRNA
ns siRNA
α4
GAPDH
C
α4-4 siRNA
α4-3 siRNA
ns siRNA
α4
GAPDH
D
LNCaP
α4-4 siRNA
MID1-3 siRNA
relative mRNA levels siRNA/ns siRNA
1,20
1,00
0,80
0,60
0,40
0,20
0,00
GAPDH
AR1
AR2
GAPDH
AR1
AR2
E
LNCaP
MID1
α4
relative mRNA levels overexpression/empty vector
1,20
1,00
0,80
0,60
0,40
0,20
0,00
GAPDH
AR1
AR2
GAPDH
AR1
AR2

Legend to supplemental Figure 1:

(A) Westernblot as knockdown control for MID1-3 and MID1-9 siRNAs shown by reduction of FLAG-tagged MID1 overexpressed in LNCaP cells, (B,C) Westernblots as knockdown-controls for the α4-3 and α4-4 siRNAs using an antibody against endogenous α4 in LNCaP cells (**D**) Real-Time PCR analysis of AR mRNA levels in LNCaP cells after knockdown of MID1 or α4 relative to the non-silencing controls. (**E**) Real-Time PCR analysis of AR mRNA levels in LNCaP cells after over-expression of MID1 or α4 relative to the control (empty vector).
